# Evaluation of the Pharmacokinetics of Intranasal Drug Delivery for Targeting Cervical Lymph Nodes in Rats

**DOI:** 10.3390/pharmaceutics13091363

**Published:** 2021-08-30

**Authors:** Tomoyuki Furubayashi, Daisuke Inoue, Shunsuke Kimura, Akiko Tanaka, Toshiyasu Sakane

**Affiliations:** 1 Department of Pharmaceutical Technology, Kobe Pharmaceutical University, 4-19-1 Motoyamakitamachi, Higashinada-ku, Kobe 658-8558, Japan; a-tanaka@kobepharma-u.ac.jp (A.T.); sakane@kobepharma-u.ac.jp (T.S.); 2 School of Pharmacy, Shujitsu University, 1-6-1 Nishigawara, Naka-ku, Okayama 703-8516, Japan; 3 College of Pharmaceutical Sciences, Ritsumeikan University, 1-1-1 Noji-higashi, Kusatsu 525-8577, Japan; d-inoue@fc.ritsumei.ac.jp; 4 Faculty of Pharmaceutical Sciences, Doshisha Women’s College of Liberal Arts, Kodo, Kyotanabe 610-0395, Japan; shkimura@dwc.doshisha.ac.jp

**Keywords:** intranasal, cervical lymph nodes, pharmacokinetic, methotrexate, targeted drug delivery

## Abstract

A well-developed lymphatic network is located under the nasal mucosa, and a few drugs that permeate the nasal mucosa are absorbed into the lymphatic capillaries. Lymph from the nasal cavity flows to the cervical lymph nodes (CLNs). In this study, we evaluated the pharmacokinetics of the direct transport of intranasally administered drugs to CLNs through the nasal mucosa of Wistar rats using methotrexate as a model drug. The drug targeting index, which was calculated based on the areas under the concentration–time curves after intravenous and intranasal administration, was 3.78, indicating the benefits of nasal delivery of methotrexate to target CLNs. The direct transport percentage, which was indicative of the contribution of the direct nose–CLN pathway of methotrexate after intranasal administration, was 74.3%. The rate constant of methotrexate from the nasal cavity to CLNs was 0.0047 ± 0.0013 min^−1^, while that from systemic circulation to CLNs was 0.0021 ± 0.0009 min^−1^. Through pharmacokinetic analysis, this study demonstrated that the direct nasal–CLN pathway contributed more to the transport of methotrexate to the CLNs than the direct blood–CLN pathway.

## 1. Introduction

In the last few decades, the intranasal (i.n.) route of administration for systemic drug delivery has received considerable attention [1]. A range of compounds, from extremely lipophilic drugs to polar, hydrophilic molecules, including peptides and proteins, have been studied for their potential applicability in i.n. administration [2,3]. The relatively high permeability of the nasal epithelium, its high vascularization in the lamina propria [4,5], and the ability to avoid hepatic first-pass metabolism render intranasal drug delivery a promising route for drug administration, especially for drugs that are metabolized in the intestine and/or liver [6]. A well-developed lymphatic network is located under the nasal mucosa, and a few drugs that permeate the nasal mucosa are distributed through the lymph capillaries. Lymph nodes are partially located on lymphatic vessels and function as filters to remove pathogens and antigens present in the lymphatic fluid.

Lymph from the oral cavity, tongue, nasal cavity, and pharynx flows into the cervical lymph nodes (CLNs). Inflammation in CLNs may develop due to infection or metastasis in the head [7,8]. In the surgical treatment of tongue or pharyngeal cancers, not only is the primary tumor removed, but CLN dissection is also performed because these lymph nodes are considered a common site for metastasis. The relationship between CLN dissection and patient prognosis is not fully understood, and there are advantages and disadvantages to dissecting nodal metastases [9]. When normal chemotherapy is used for the treatment of CLN metastasis, care should be exercised to minimize the systemic side effects of antitumor drugs. A preliminary study conducted by our group suggested that intranasal administration of a model drug might enable direct delivery to the CLNs, thereby minimizing systemic side effects. Furthermore, the nasal mucosa presents with well-developed lymphatic tissue, which serves as the first line of defense against microbial invasion [7]. It also functions as a site for the application of agents for immunization against influenza [8]. If immunostimulatory agents, such as peptide vaccines for cancer, are administered nasally, such that they can be delivered directly to the CLNs, efficient immunization against cancer may be achieved. Although targeting of drugs to the CLNs and the nasal immune system may be considered for the treatment of various diseases, there have been a few reports published on i.n. drug administration for targeting the CLNs. Ross et al. [10] reported that interferon beta was delivered directly to the central nervous system and CLNs via i.n. administration. However, they have not analyzed the direct delivery to CLNs based on pharmacokinetics. 

In this study, we evaluated the pharmacokinetic profile of intranasally administered methotrexate (MTX) as a model drug in terms of its ability to be directly transported to the CLNs through the nasal mucosa in rats. MTX is an anticancer drug useful for the treatment of several malignancies, such as leukemia, hematologic malignancies, osteosarcoma, breast and cervical cancer, and rheumatoid arthritis [11,12]. Since the bioavailability after i.n. administration in rats was found to be extremely high (98.2%) [13] in our previous study, and the gastrointestinal absorption of MTX was negligible, MTX was deemed a suitable model to evaluate direct transport to the CLNs.

## 2. Materials and Methods

### 2.1. Materials

MTX was purchased from Santa Cruz Biotechnology, Inc. (Santa Cruz, CA, USA). Phosphate-buffered saline (pH 7.4) was purchased from Nacalai Tesque Co. (Kyoto, Japan). All other chemicals were of reagent grade and were commercially available. 

Eight-week-old male Wistar rats were purchased from Japan SLC, Inc. (Shizuoka, Japan). All animal studies were approved by the Committee of the Animal Care of Shujitsu University and were conducted according to the guidelines (Approval ID: 013-002, 25 May 2015). Rats were housed under controlled temperatures of 20–26 °C and 40–60% humidity, with a 12-h light/dark cycle. The animals were fed ad libitum until the day prior to the day of conduction of in vivo experiments.

### 2.2. Intravenous and Intranasal Drug Administration in Rats 

Under anesthetization conditions, the right femoral artery was cannulated with polyethylene tubing (SP-31, Natsume, Tokyo, Japan) to collect blood samples, and rats fixed in the supine position received intravenous (i.v.) or i.n. administration of MTX (0.05 mg/rat). MTX solution (0.2 mL) was injected into the left femoral vein using a 1 mL syringe with a 26G needle, and MTX dissolved in 5 µL of physiological saline was instilled rapidly at a 1 cm depth from the nostril using a 10 µL microsyringe. To obtain the plasma MTX concentration–time profiles following i.v. and i.n. administration, blood samples (ca. 0.5 mL) were collected intermittently in heparinized tubes at 1, 3, 5, 10, 20, 30, 60, 90, 120, and 180 min after i.v. (*n* = 4) and i.n. (*n* = 6) administration in the supine position, and samples were centrifuged to separate plasma (3000 rpm, 10 min). Methanol (1.2 mL) was added to the plasma samples (0.1 mL) for deproteinization, and the mixture was centrifuged. For the CLN MTX concentration–time profiles, four lymph nodes, two each from the left and right sides of a rat, were isolated at each sampling point (10, 30, 60, 90, 120, and 180 min) after i.v. (*n* = 4; each sampling point) or i.n. (*n* = 6; each sampling point) administration and were homogenized together using 2 mL chilled methanol for deproteinization and MTX extraction.

The homogenate was centrifuged to separate the supernatant (15,000 rpm, 10 min). The concentrations of MTX in plasma and CLN samples were determined via liquid chromatography–mass spectrometry (LC/MS) analysis. Some blood vessels were observed to be present in the CLNs; therefore, the concentration of MTX in CLNs was determined by subtracting the value for the amount of MTX in blood from that of the total amount in the CLNs, as determined by LC/MS (Equation (1)).

(1) $$\mathrm{Accurate}\;\mathrm{amount}\;\mathrm{of}\;\mathrm{MTX}\;\mathrm{in}\;\mathrm{CLN}=X_{\mathrm{CLN},\mathrm{obs}}-\left(C_{\mathrm{plsm}}\times V_{\mathrm{vsl},\mathrm{CLN}}\right)$$

Here, *X*_CLN,obs_ represents the amount of MTX in the CLNs, *C*_plsm_ represents the concentration of MTX in plasma at the time of CLN collection, and *V*_vsl,CLN_ represents the volume of blood vessels in the CLNs. The blood vessel volume in the CLNs was determined by measuring the radioactivity of [^3^H]-inulin in the CLNs and blood samples of rats 2 min after bolus i.v. administration (Equation (2)).

(2) $$\mathrm{Volume}\;\mathrm{of}\;\mathrm{blood}\;\mathrm{vessels}\;\mathrm{in}\;1\;g\;\mathrm{of}\;\mathrm{CLN}=\frac{\mathrm{Lymph}\;\left(\mathrm{dpm}/g\;\mathrm{CLN}\right)}{\mathrm{Plasma}\;\left(\mathrm{dpm}/\mathrm{mL}\right)}\;=0.1047\pm 0.0073\;\left(\mathrm{mL}/g\;\mathrm{CLN}\right)\;\;\;\left(s.e.,\;n=16\right)$$

### 2.3. Assay

Plasma and CLN samples from the in vivo study were analyzed for MTX concentration via mass spectrometry using an LC/MS system (APL1100, Agilent Technology, Palo Alto, CA, USA) equipped with a reversed-phase column (Inertsil ODS-3, 2.1 × 150 mm, GL Sciences Inc., Tokyo, Japan). The mobile phase comprised 50 mM HCOONH_4_:MeOH (70:30 *v*/*v*). A flow rate of 0.2 mL/min and an injection volume of 20 µL were considered. The conditions for mass spectrometry were as follows: drying gas temperature, 350 °C; drying gas flow, 13.0 L/min; nebulizing gas pressure, 54 psig; capillary voltage, 3000 V. The mass spectrometer was operated in the positive-ion mode ([M + H]^+^), with a fragmenter voltage of 120 V. The protonated MTX molecule presented with an m/z value of 455.2. The peak area of MTX showed a good correlation with its concentration over a wide range (1–500 ng/mL), with an R^2^ higher than 0.9992, indicating that concentrations within this range could be precisely determined.

### 2.4. Calculation of the Area under the Concentration–Time Curve (AUC) and Mean Resistance Time (MRT)

The plasma/CLM MTX concentration–time curves for the period from 0 to 180 min after drug administration were plotted using the data on average concentrations at each time point, and *AUC* (*AUC*_plsm_ or *AUC*_CLN_) was calculated using the trapezoidal rule. Each *AUC* for the period from 0 to infinity was calculated by adding *AUC* obtained for the period from 0 to 180 min and AUC obtained for the period from 180 to infinity after i.v. or i.n. administration. The *AUC* obtained for the period from 180 min to infinity was defined as the concentration of MTX at 180 min divided by the elimination rate constant. The elimination rate constant was calculated based on the elimination phase (90, 120, and 180 min) of the curve, assuming negligible influx from the nasal cavity and circulating blood, because Akaike’s information criteria (AIC) calculated using a computer program for nonlinear regression analysis helped clarify that the use of a monoexponential equation was better for expressing the elimination of MTX than the use of a biexponential equation.

The *MRT* for plasma or CLN (*MRT*_plsm_ or *MRT*_CLN_) after i.v. and i.n. administration was calculated using the *AUC*_plsm_ or *AUC*_CLN_ and the area under the moment curve (*AUMC*_plsm_ or *AUMC*_CLN_) according to Equation (3) as follows:

(3) $$MRT=\frac{{\left[AUMC_{\mathrm{plsm}\;\mathrm{or}\;\mathrm{CLN}}\right]}_{i.v.\;\mathrm{or}\;i.n.}}{{\left[AUC_{\mathrm{plsm}\;\mathrm{or}\;\mathrm{CLN}}\right]}_{i.v.\;\mathrm{or}\;i.n.}}=\frac{{\left[\int_{\infty }^{0}t\;\cdot \;C_{\mathrm{plsm}\;\mathrm{or}\;\mathrm{CLN}}\;dt\right]}_{i.v.\;\mathrm{or}\;i.n.}}{{\left[\int_{\infty }^{0}C_{\mathrm{plsm}\;\mathrm{or}\;\mathrm{CLN}}\;dt\right]}_{i.v.\;\mathrm{or}\;i.n.}}$$

### 2.5. Calculation of Drug Targeting Index (DTI) and Direct Transport Percentage (DTP) 

The degree of drug targeting to the CLNs after i.n. administration was evaluated using *DTI*, which was calculated as per methods described by Wang et al. [14]. To understand the extent of nose-to-brain targeting, the ratio of *AUC*_CLN_/*AUC*_plsm_ after i.n. administration to *AUC*_CLN_/*AUC*_plsm_ after i.v. administration was calculated as per the formula shown in Equation (4):

(4) $$DTI=\frac{\frac{AUC_{\mathrm{CLN},\;i.n.}}{AUC_{\mathrm{plsm},\;i.n.}}}{\frac{AUC_{\mathrm{CLN},\;i.v.}}{AUC_{\mathrm{plsm},\;i.v.}}}$$

Furthermore, to evaluate the contribution of the direct nose–CLN pathway, the *DTP* was calculated using the following expression, as reported by Zhang et al. (Equation (5)) [15]:

(5) $$DTP\left(\%\right)=\frac{AUC_{\mathrm{CLN},\;i.n.}-AUC_{\mathrm{CLN},\;i.v.}\cdot \;\frac{AUC_{\mathrm{plsm},\;i.n.}}{AUC_{\mathrm{plsm},\;i.v.}\;}}{AUC_{\mathrm{CLN},\;i.n.}}\times 100$$

In both above-mentioned equations, *AUC*_CLN,i.n._ and *AUC*_CLN,i.v._ represent the *AUC*s of MTX in the CLNs after i.n. and i.v. administration, respectively, while *AUC*_plsm,i.n._ and *AUC*_plsm,i.v._ represent the *AUC*s of MTX in plasma after i.n. and i.v. administration, respectively.

### 2.6. Analysis of Pharmacokinetic Parameters

**Figure 1:**
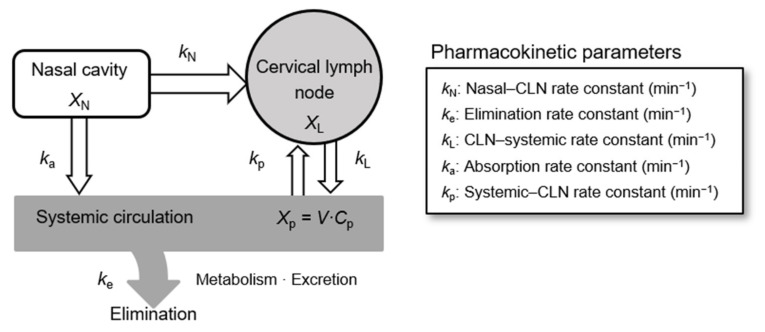
Model illustrating drug absorption and disposition following nasal administration and associated pharmacokinetic parameters. Abbreviations: CLN, cervical lymph node; *X*, mass of methotrexate in each compartment; *k*, rate constant; *V*, volume of distribution; *C*, concentration of methotrexate; N, nasal cavity; L, cervical lymph node; p, plasma.

**Figure 2:**
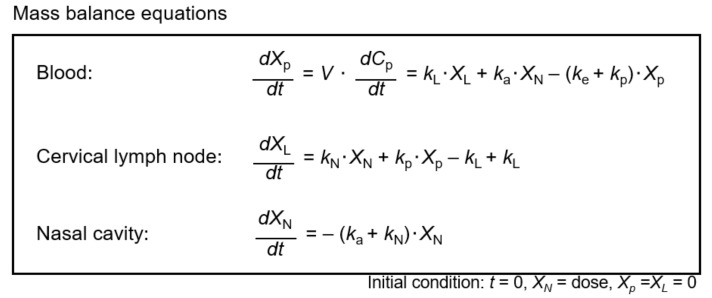
Mass balance equations used for the calculation of pharmacokinetic parameters based on the absorption–disposition model considered for nasal administration. Abbreviations: *X*, mass of methotrexate in each compartment; *k*, rate constant; *V,* volume of distribution; *C*, concentration of methotrexate; N, nasal cavity; L, cervical lymph node; p, plasma.

The model for drug absorption and elimination following i.n. administration and the associated pharmacokinetic parameters are shown in Figure 1. Pharmacokinetic parameters were determined based on the mass balance equations shown in Figure 2, using noncompartmental and compartmental techniques. The analysis was conducted using WinNonlin version 6.3 (Certara, Princeton, NJ, USA).

### 2.7. Statistical Analysis

Experiments for determination of CLN MTX concentration–time profiles following i.v. and i.n. administration were performed using three and six rats for each sampling point, respectively, and the results have been expressed as mean ± standard error. Statistical significance was determined using Student’s *t*-test at *p* < 0.05.

## 3. Results and Discussion

### 3.1. Intravenous and Intranasal Administration of MTX in Rats 

**Figure 3:**
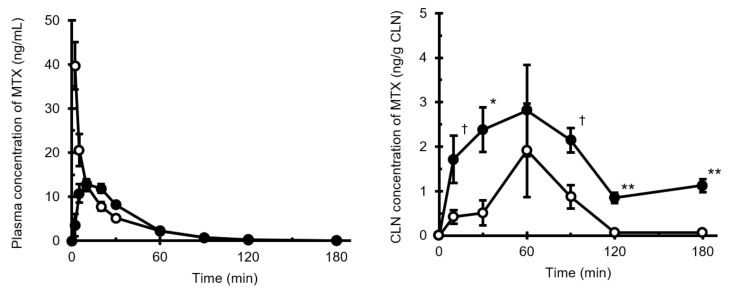
Plasma (left) and CLN (right) MTX concentration–time profiles following intranasal and intravenous administration. Key: ○, i.v. administration; ●, i.n. administration. Data points have been expressed as mean ± standard error for four (i.v.) and six (i.n.) experiments at each time point in the plasma concentration–time profile and for four (i.v.) and six (i.n.) experiments at each time point in the CLN concentration–time profile. ^†^ *p* < 0.10, * *p* < 0.05, ** *p* < 0.01. Abbreviations: CLN, cervical lymph node; MTX, methotrexate.

The plasma and CLN MTX concentration–time profiles following i.n. and i.v. administration are shown in Figure 3. MTX was rapidly absorbed into the systemic circulation following i.n. administration, and the time to reach maximum plasma concentration (*T*_max,plsm_) was 10 min. *AUC*_plsm_ values after i.v. (*AUC*_plsm,i.v._) and i.n. (*AUC*_plsm,i.n._) administration of MTX were 554.9 ng·min/mL and 536.8 ng·min/mL, respectively. The absolute bioavailability, which was calculated by dividing *AUC*_plsm,i.n._ by *AUC*_plsm,i.v._, was found to be 0.967 (Table 1). 

In contrast, the CLN concentration of MTX following i.n. administration increased more rapidly than that following i.v. administration, and the increase in MTX concentration persisted for up to 60 min. A time lag was observed between the time to the maximum concentration of CLN (*T*_max,CLN_) and *T*_max,plsm_. These results indicated that the transport of MTX through the nasal mucosa to the CLNs might be slower than that through systemic circulation. *AUC*_CLN_ values obtained for the period from 0 to 180 min after i.v. (*AUC*_CLN,i.v._) and i.n. (*AUC*_CLN,i.n._) administration were 129.9 and 491.0 ng·min/g CLN, respectively (Table 1). *AUC*_CLN,i.n._ was found to be 4.02-fold higher than *AUC*_CLN,i.v._ The *MRT*_plsm_ and *MRT*_CLN_ after i.n. administration were longer than those obtained after i.v. administration. These results indicate that the efflux of MTX from the cervical lymph into the systemic circulation may be slower than the elimination of MTX from systemic circulation, which is consistent with the findings reported by Sudo et al. [16], based on their study of dopamine levels in the lymph from the cervical lymph trunk following i.v. bolus injection. They observed that the cervical lymph flow in rats was approximately 3 µL/min/kg; in the present study, the efflux of MTX from CLNs seemed to be slower in comparison. 

The *DTI*, which was calculated using the *AUC* values, was 3.78 (Table 1), indicating the benefits of i.n. delivery in targeting CLNs. Serralheiro et al. [17] reported that *DTI* could be used as an index of the predominance of the i.n. route; the transport of drugs to the target tissue is predominant when *DTI* is greater than 1. Additionally, the *DTP*, considered an indicator of the contribution of the direct nose–CLN pathway in CLN targeting of drugs following i.n. administration, was found to be 74.3%. These findings suggest that the direct nasal–CLN pathway contributes more significantly to the transport of MTX to CLNs than the direct blood–CLN pathway.

The *AUC*s were calculated using the average concentrations obtained at each time point according to the trapezoidal rule. Other parameters were calculated using the obtained *AUC*s according to the equations described in the text.

### 3.2. Analysis of Pharmacokinetic Parameters

**Figure 4:**
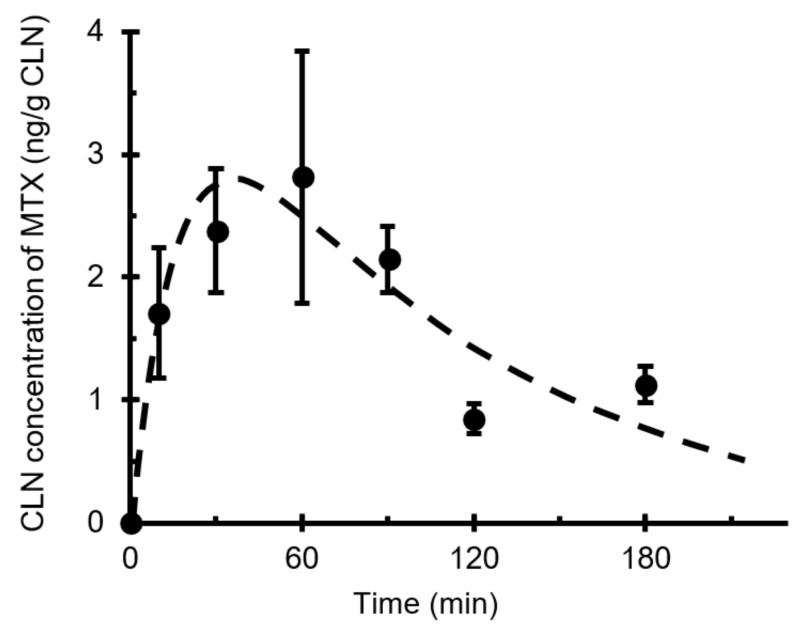
Pharmaceutical model CLN concentration–time curve of intranasally administered MTX fit to experimental data. Key: ●, concentration in the CLN; - - - -, fitted profile. Curve fitting was performed via nonlinear regression using WinNonlin version 6.3. Data points are expressed as mean ± standard error values of at least four experiments. Abbreviations: CLN, cervical lymph node; MTX, methotrexate.

The curve fit illustrated in Figure 4 was obtained using mass balance equations (Figure 2) and the pharmacokinetic absorption–elimination model (Figure 1). The rate constant (*k*_N_) of MTX from the nasal cavity to the CLNs (direct nose–CLN pathway) was 0.0047 ± 0.0013 min^−1^, while the rate constant (*k*_p_) based on the systemic circulation to the CLN (direct blood–CLN pathway) was 0.0021 ± 0.0009 min^−1^ (Table 2). Therefore, the delivery of MTX to CLNs via the nasal cavity was faster than that through systemic circulation. The relationship between *k*_N_ and *k*_p_ is similar to the quantitative relationship described in the preceding section. It was found that i.n. administration of MTX showed predominance over i.v. administration regarding delivery to the CLNs. It is assumed that the low molecular weight of MTX promotes easy transfer from the submucosal layer to the blood vessels, whereas molecular weights exceeding 5000 are generally advantageous for transport of molecules from the subcutaneous or submucosal layer to lymphatic vessels [18]. Additionally, the superficial network of lymphatic capillaries, which is located at almost the same depth [19,20], is not well situated for small-molecule drug distribution compared to the network of blood capillaries that are present close to the epithelium. Although the transport of MTX from the submucosal layer to the CLN is not easy, it is predominant during i.n. administration. Consequently, it was revealed that i.n. administration was particularly effective for drugs with low transport efficiency from systemic circulation to CLNs. 

Furthermore, the elimination rate constants of MTX deduced from systemic circulation and CLN–systemic circulation rate constants were 0.0737 ± 0.0208 min^−1^ and 0.0095 ± 0.0039 min^−1^, respectively. A possible explanation for the slow elimination of MTX from CLNs may be put forth based on the slow rate of cervical lymph flow (approximately 3–4 μL/min) [16]. This implies that once the drug reaches the CLN, effusion of the drug is difficult. The quantitative relationships and pharmacokinetic analyses suggest that i.n. administration will help facilitate the maintenance of an adequate concentration of MTX in CLNs, while suppressing systemic side effects, since its plasma concentration decreases rapidly. 

Furthermore, the CLN concentration of the drug may be increased via repeated administration. In the treatment of diseases, such as metastasis to the CLNs, it may be necessary to increase the drug concentration in CLNs or to prolong the transport time from the nasal cavity. These findings suggest that i.n. administration is an effective strategy for drug targeting to CLNs.

It is important to ascertain whether the results in rats are applicable to humans. Johnston et al. [21] have demonstrated that there are extensive lymphatic networks in the submucosa associated with the olfactory and respiratory epithelia in humans and rats. Although the nasal mucosal surface area and the ratio of olfactory and respiratory regions differ considerably between rats and humans, the fact remains that the lymph network undergoes extensive development throughout the nasal mucosa, and observations of mucociliary clearance, which leads to the removal of various types of xenobiotics, such as viruses, drugs, and dust, are almost identical in rats and humans [22]. Therefore, the authors suggest MTX may also be delivered to the CLN in humans.

## 4. Conclusions

This study demonstrated the predominance of the i.n. route for drug delivery to the CLN in rats through pharmacokinetic analysis because MTX was found to be delivered efficiently to the CLNs via i.n. administration. Drugs that possess physicochemical properties similar to those of MTX, such as molecular weight and permeability, may be delivered efficiently to the CLN via i.n. administration, suggesting that this route of administration may be useful for the delivery of antitumor drugs or peptides for the treatment of metastasis and cancers associated with CLNs.

## Figures and Tables

**Table 1:** Areas under the concentration–time curves and associated parameters following intravenous and intranasal administration.

	i.v.	i.n.
*AUC*_plsm_ (ng·min/mL)	554.9	536.8
*AUC*_CLN_ (ng·min/g CLN)	129.9	491.0
*F* (%)		96.7
*MRT*_plsm_ (min)	24.7	31.1
*MRT*_CLN_ (min)	56.6	146.1
*DTI*		3.78
*DTP* (%)		74.3

Abbreviations: *AUC*, area under the plasma concentration–time curve; *AUC_CLN_*, area under the cervical lymph node concentration–time curve; *F*, bioavailability; *MRT*, mean residence time; *DTI,* drug targeting index; *DTP*, direct transport percentage.

**Table 2:** Rate constants calculated using WinNonlin.

Rate Constants	min^−1^
*k*_e_	0.0740 ± 0.0208
*k*_a_	0.1014 ± 0.0211
*k*_N_	0.0047 ± 0.0013
*k*_L_	0.0095 ± 0.0039
*k*_p_	0.0021 ± 0.0009

The rate constants were determined based on mass balance equations (Figure 2) by adopting noncompartmental and compartmental techniques using the WinNonlin version 6.3 software. Abbreviations: *k*_e_, elimination rate constant; *k*_a_, absorption rate constant; *k*_N_, nasal–cervical lymph node transport rate constant; *k*_L_, cervical lymph node–systemic circulation transport rate constant; *k*_p_, systemic circulation–cervical lymph node transport rate constant.

## Data Availability

The data presented in this study are available on request from the corresponding author.
